# International practice patterns in the management of pouchitis: results from a global survey

**DOI:** 10.1093/crocol/otag114

**Published:** 2026-10-12

**Authors:** Fabrizio Fanizzi, Ferdinando D’Amico, Maria Galbiati, Vipul Jairath, Laurent Peyrin-Biroulet, Silvio Danese

**Affiliations:** IRCCS Ospedale San Raffaele, Department of Gastroenterology and Endoscopy, 20132, Milan, Italy; Faculty of Medicine and Surgery, Vita e Salute San Raffaele University, 20132, Milan, Italy; IRCCS Ospedale San Raffaele, Department of Gastroenterology and Endoscopy, 20132, Milan, Italy; IRCCS Ospedale San Raffaele, Department of Gastroenterology and Endoscopy, 20132, Milan, Italy; Faculty of Medicine and Surgery, Vita e Salute San Raffaele University, 20132, Milan, Italy; Department of Epidemiology and Biostatistics, Western University, London, Ontario, N6G 2M1, Canada; Division of Gastroenterology, Department of Medicine, Western University, London, Ontario, N6G 2M1, Canada; Department of Gastroenterology, INFINY Institute, INSERM NGERE, CHRU Nancy, F-54500 Vandœuvre-lès-Nancy, France; IRCCS Ospedale San Raffaele, Department of Gastroenterology and Endoscopy, 20132, Milan, Italy; Faculty of Medicine and Surgery, Vita e Salute San Raffaele University, 20132, Milan, Italy

**Keywords:** pouchitis, ileal pouch–anal anastomosis, IPAA, pouch, inflammatory bowel disease, survey

## Abstract

**Background:**

Pouchitis is the most frequent long-term complication after ileal pouch–anal anastomosis (IPAA) for ulcerative colitis. This study aimed to describe current international practices, definitions, and treatment strategies among experienced clinicians.

**Methods:**

An international web-based survey was conducted in 2025, following CROSS guidelines, exploring diagnostic, monitoring, and therapeutic practices. Responses were anonymized and analyzed descriptively.

**Results:**

A total of 412 physicians from 49 countries completed the survey, mostly gastroenterologists involved in IBD care (362/412, 87.8%). Most clinicians (156/412, 37.9%) estimated a pouchitis prevalence of 26%-50% among patients with IPAA, typically occurring within the first postoperative year. Follow-up was scheduled every 3-6 months by 164/412 (39.8%) physicians, with nearly half (47.6%) reporting the use of the pouch disease activity index (PDAI). Fecal calprotectin was the preferred non-invasive monitoring tool (326/412, 79.1%), whereas intestinal ultrasound was selected by 130/412 respondents (31.69%). Endoscopy was the primary diagnostic and follow-up modality (398/412, 96.6%), with biopsies routinely taken (364/412, 88%), although 46.7% of respondents reported not using a standardized histological scoring system. Prophylactic therapy was prescribed by 222/412 (53.9%), mainly probiotics (27.7%) and rectal mesalazine (18.2%). In acute pouchitis, ciprofloxacin plus metronidazole was the preferred first-line regimen (218/412, 52.9%). For chronic antibiotic-dependent or refractory pouchitis, advanced therapies were the predominant first-line option (254/412, 61.7%), with vedolizumab (198/412, 48.1%), and infliximab (170/412, 41.3%) most frequently selected. Ustekinumab, selective IL-23 inhibitors, JAK inhibitors and S1P receptor modulators were increasingly used in refractory cases. Treatment targets were primarily clinical (156/412, 37.9%) and endoscopic (124/412, 30.1%) remission, and most respondents (246/396, 62.1%) reported performing endoscopic assessment within 3-6 months after advanced treatment initiation.

**Conclusions:**

This international survey demonstrates substantial heterogeneity in pouchitis diagnosis, monitoring, and treatment, highlighting important gaps in standardized disease assessment and treatment strategies. The findings support the need for consensus-based approaches and prospective, pouch-specific studies to improve the standardization of pouchitis care.

## Teaser

Short summary

This international survey of 412 clinicians reveals wide heterogeneity in pouchitis diagnosis, monitoring, and treatment after IPAA, with increasing use of advanced therapies, limited standardized scoring, and urgent need for consensus definitions and treatment algorithms to improve patient outcomes.

Key Messages

- **What is already known?** Pouchitis is the most frequent long-term complication after IPAA for ulcerative colitis, with antibiotics as first-line therapy and no universally accepted standards for diagnosis, monitoring or treatment.
- **What is new here?** This 2025 international survey shows wide heterogeneity in pouchitis definitions, management and treatment.
- **How can this study help patient care?** By identifying major variations in practice, this study supports the development of standardized diagnostic criteria and treatment algorithms to improve consistency and outcomes in pouchitis care.

## Introduction

Pouchitis represents the most frequent complication following ileal pouch–anal anastomosis (IPAA), a restorative surgical procedure commonly performed for patients with ulcerative colitis (UC) requiring colectomy. Pouchitis occurs in approximately 48% of patients within the first 2 years after surgery^1^ and up to 80% of patients experience pouchitis symptoms at some point during follow-up.^2^^,^^3^ The clinical presentation ranges from mild, antibiotic-responsive inflammation to chronic, refractory disease associated with significant morbidity and impaired pouch function.^1^^,^^4^ Pouchitis can be broadly classified into 4 clinical phenotypes. Intermittent pouchitis refers to infrequent, self-limiting episodes that typically resolve spontaneously or with short antibiotic courses.^5^^,^^6^ Chronic antibiotic-dependent pouchitis (CADP) responds to antibiotics but tends to relapse soon after discontinuation.^5^^,^^6^ Chronic antibiotic-refractory pouchitis (CARP) fails to respond to antibiotics and generally requires corticosteroids or advanced therapy.^5^^,^^6^ Finally, Crohn’s-like disease of the pouch is defined by features such as late-onset perianal, fistulas (≥12 months after IPAA), strictures of the pouch body or pre-pouch ileum, and pre-pouch ileitis.^7^ Over the past 2 decades, considerable progress has been made in understanding the pathogenesis of pouchitis. Dysbiosis, mucosal immune dysregulation, and residual inflammatory activity are recognized as key contributors to disease development.^8^^,^^9^ Nevertheless, clinical practice remains heterogeneous, largely due to the absence of standardized diagnostic criteria and evidence-based management strategies.^10^^,^^11^ Antibiotics, particularly ciprofloxacin and metronidazole, remain the cornerstone of therapy for acute pouchitis.^5^^,^^11^ In contrast, patients with chronic antibiotic-dependent and antibiotic-refractory pouchitis represent more challenging clinical scenarios, for which therapeutic strategies are less clearly defined. Advanced therapies, including biologics and small molecules, are increasingly employed in these cases, although their optimal positioning within treatment algorithms has yet to be standardized.^10^^,^^12^ Moreover, preventive and monitoring approaches are considered important adjuncts to care, while the management of pouch-related complications or pouch failure requires an individualized, multidisciplinary strategy. Persistent uncertainty regarding prevention, long-term monitoring and the best approach to complex cases underscores the need for consensus and evidence-based guidelines.^10^ While several single-center studies and expert reviews have described local practices, comprehensive data on global trends in pouchitis management are lacking. Understanding international variations in clinical practice is essential to identify unmet needs and to inform the development of future consensus guidelines.^13^ This study aims to address this gap through a large, international survey involving physicians experienced in inflammatory bowel disease (IBD) care. Specifically, we sought to describe current diagnostic and therapeutic approaches to pouchitis across diverse healthcare settings, evaluate the use of antibiotics, probiotics, and advanced therapies, and explore definitions and treatment targets applied by physicians worldwide. By capturing real-world perspectives, this survey provides an updated insight of global clinical practice and highlights key areas where harmonization and further research are needed.

## Materials and methods

### Study design and participants

This cross-sectional, web-based survey was designed to investigate current practices in the diagnosis and management of pouchitis among clinicians involved in IBD care. The study was conducted and reported in accordance with the *Consensus-Based Checklist for Reporting of Survey Studies (CROSS)* guidelines.

The survey was distributed internationally using the SurveyMonkey platform between March and October 2025. Invitations were disseminated through professional networks, international IBD societies, and academic mailing lists to ensure broad global participation. Eligible participants included physicians actively managing patients with IBD and IPAA like gastroenterologists, surgeons and internists. Participation was voluntary and anonymous, and informed consent was obtained at the beginning of the questionnaire. Because the survey was disseminated through multiple professional networks and mailing lists, a reliable denominator for the total number of individuals who received an invitation could not be established; therefore, a global response rate could not be calculated. The final analysis included 412 respondents who provided survey responses.

### Survey structure

The questionnaire comprised 43 multiple-choice questions grouped into 5 thematic sections: demographics and professional background; diagnostic and monitoring practices; therapeutic approaches; use of advanced therapies; outcomes and complications. All responses were recorded anonymously, and duplicate entries were prevented by restricting one submission per email address.

### Data management and ethics

Responses were collected and stored securely within the SurveyMonkey platform. No imputation of missing responses was performed. Because several questions were conditional and displayed according to responses to previous items, unanswered fields could reflect either survey skip logic or non-response to an individual item. Accordingly, each survey question was analyzed using the number of respondents providing a valid answer to that specific item, and denominators are reported throughout the Results and tables where applicable. As the study was non-interventional and did not involve patient data, formal ethics approval was not required according to international standards and institutional policies. The study adhered to the principles of the Declaration of Helsinki.

### Statistical analysis

Descriptive statistics were used to summarize categorical variables as counts and percentages. Results are presented with the total number of respondents per question to account for missing data. Exploratory subgroup analyses were performed to assess whether selected diagnostic and monitoring practices varied according to IPAA caseload and the availability of a dedicated pouch clinic.

## Results

### Demographics and professional background

A total of 412 physicians participated in the survey, including 362 gastroenterologists, 30 general surgeons, 16 internal medicine specialists, 2 colorectal surgeons, and 2 pediatric gastroenterologists. The participants represented 49 countries across all continents. Most respondents were based in Europe, accounting for over two-thirds of the sample, with Italy contributing nearly one-third of all participants. Additional substantial contributions came from North and South America, particularly Argentina, Brazil, Canada, and the United States. Further global representation was provided by Asia, the Middle East, and Oceania (predominantly Australia), while a smaller proportion of respondents originated from Africa, mainly Algeria, Tunisia, Egypt, and Morocco (Figure 1).

**Figure 1:**
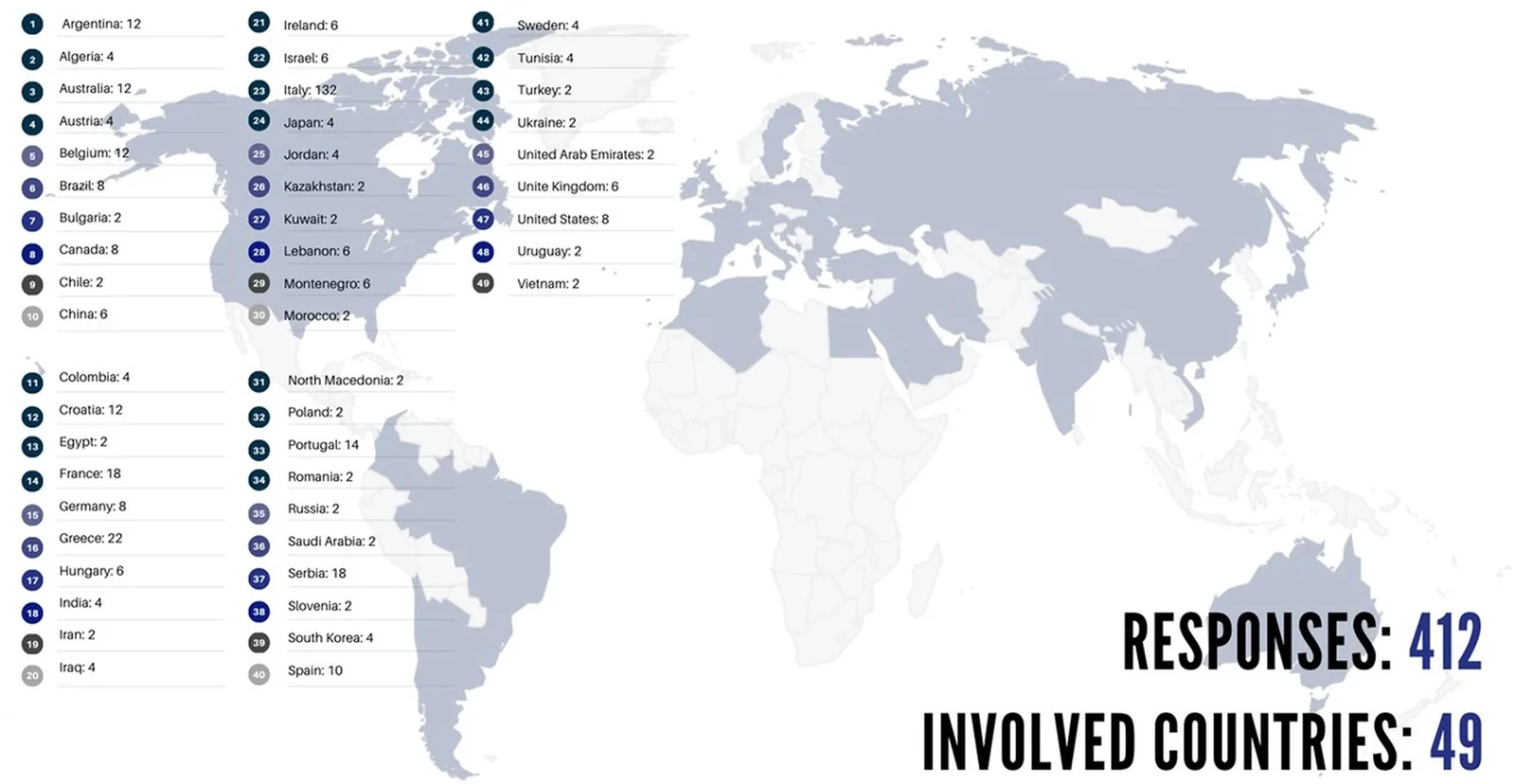
Geographic distribution of survey participants.

Almost half of the respondents were employed in university hospitals (44.2%), while a similar proportion worked in public hospitals (42.7%), reflecting the strong participation of physicians from academic and government institutions. Most physicians reported a long-standing medical career, with nearly half having more than 20 years since obtaining their medical degree. When focusing on IBD expertise, the majority of participants (68.9%) had more than 10 years of experience in the field, whereas only a very small proportion (2.4%) were early-career physicians with less than 1 year of IBD experience. Regarding clinical activity, 16% reported to manage fewer than 100 IBD patients per year while 33.5% up to 500 patients. Conversely, 25.2% followed up to 1000 patients, 18.5% more than 1000 patients annually, and 6.8% reported caseloads exceeding 2000 patients (Figure 2). When asked about their experience with patients with IPAA, most respondents reported caring for relatively few such patients annually. Specifically, 38.4% reported to manage fewer than 10 pouch patients per year and 42.2% between 10 and 50, while only 6.3% reported managing more than 100. Similarly, only a small proportion (8.3%) reported more than 100 procedures of restorative proctocolectomy annually. Most hospitals were low-volume centers, with 61.7% of participants reporting fewer than 10 IPAA surgeries per year and 26.2% reporting between 10 and 50 procedures per year. Dedicated pouch clinics were available in a minority of centers (24.8%), reflecting the limited formalization of multidisciplinary care for pouch disorders. Among centers with a specialized clinic, patient management was most frequently shared between gastroenterologists and surgeons (54.6%), while 31.5% reported gastroenterologist-led care and only 4.6% surgeon-led care (Figure 3).

**Figure 2:**
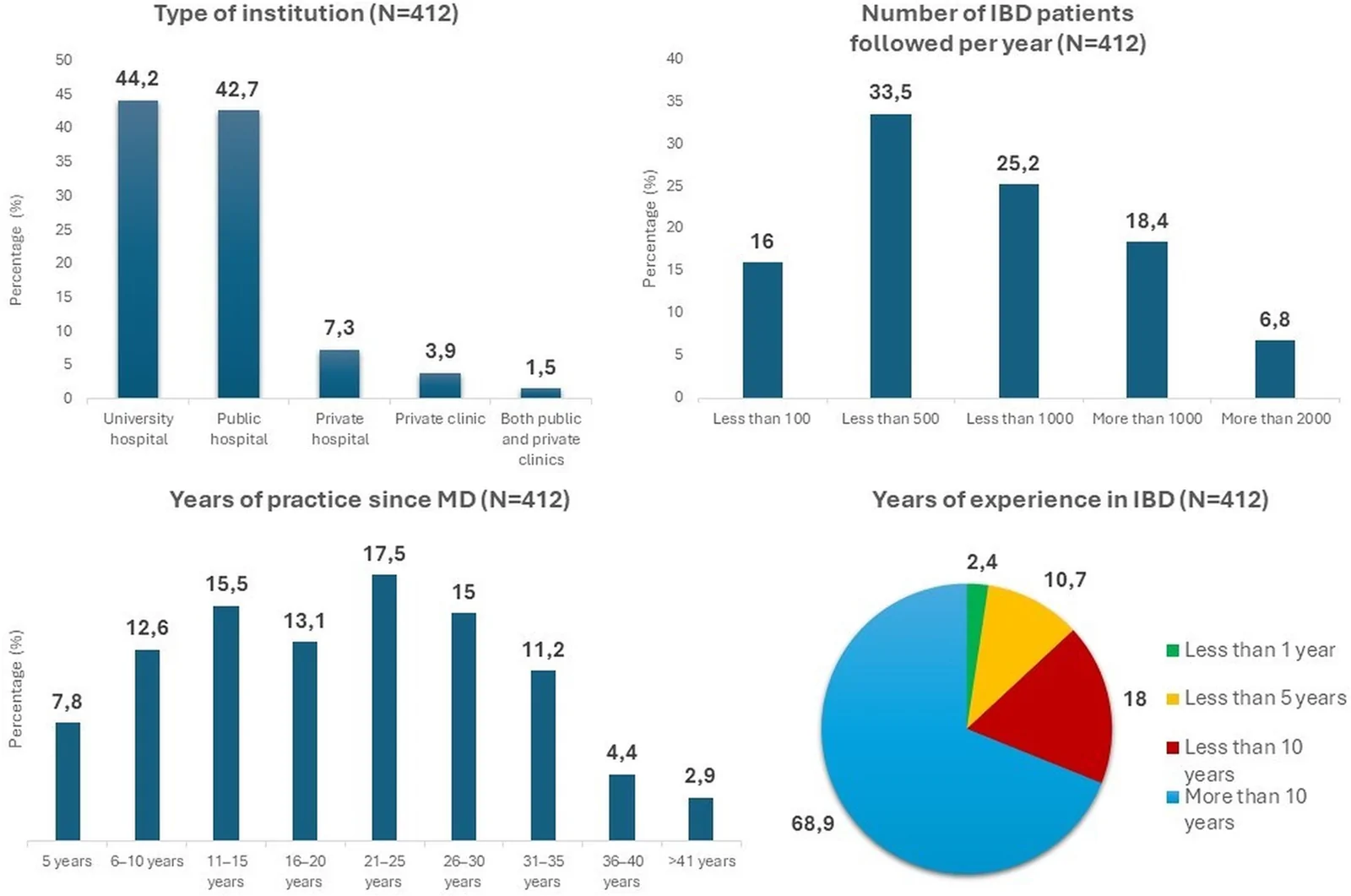
Professional background and clinical practice characteristics of survey respondents, with a focus on IBD and IPAA management (*N* = 412)..

**Figure 3:**
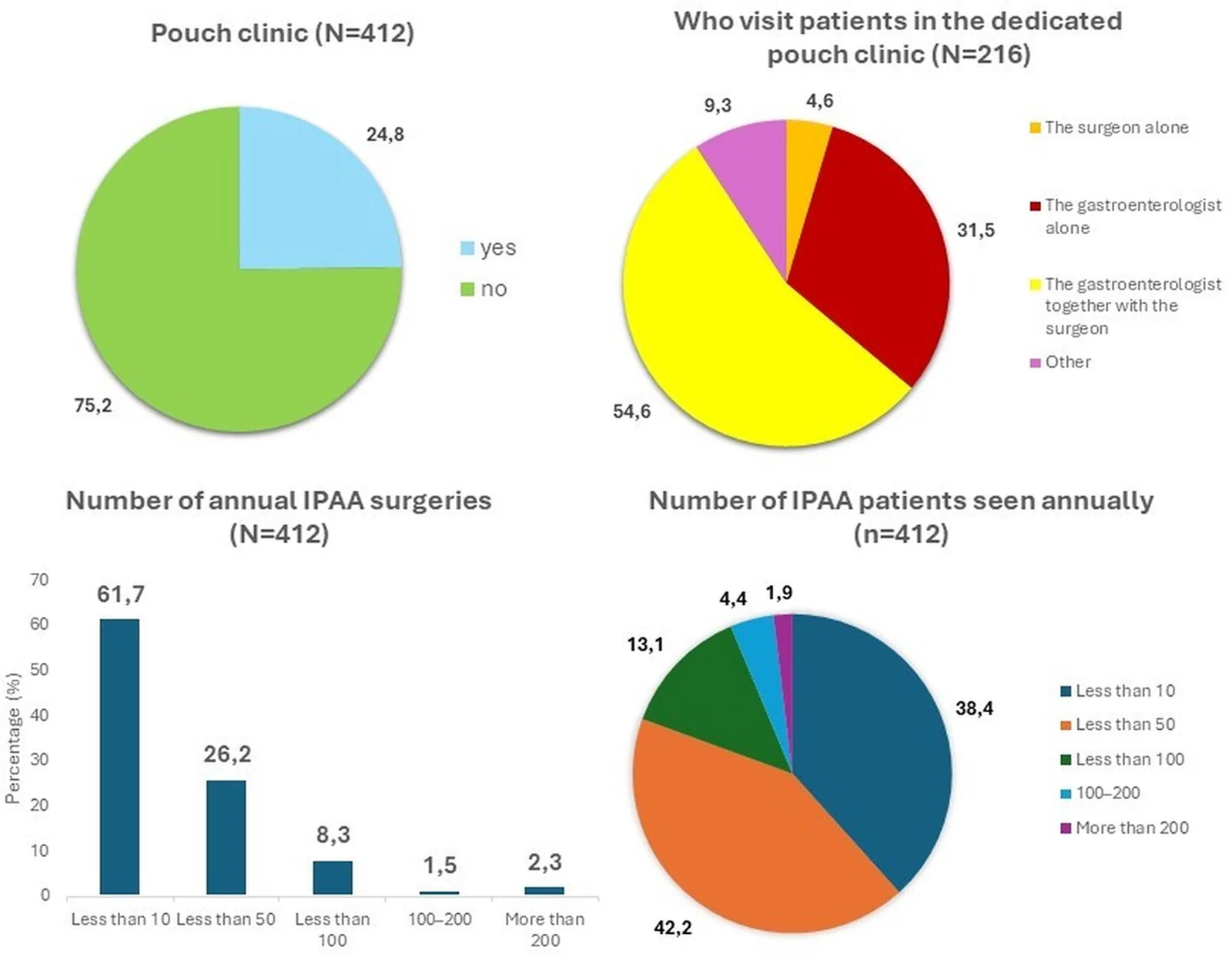
Professional background and clinical practice characteristics of survey respondents, with a focus on IBD and IPAA management (*N* = 412).

### Diagnostics and monitoring practices in IPAA patients

Most respondents estimated a pouchitis prevalence of 26%-50% among their patients (37.9%), while nearly one-third (29.1%) reported lower rates (11%-25%), and a smaller proportion (18.4%) estimated a prevalence below 10%. Pouchitis was most commonly reported between 4 and 12 months after surgery (60.2% overall: 26.7% at 4-6 months, 33.5% at 7-12 months), suggesting that the perceived risk is highest within the first postoperative year. Nevertheless, about one-third of clinicians (32.0%) observed onset beyond 12 months. Follow-up was generally scheduled every 3-6 months (71.8%), although considerable variation was observed across respondents (Table 1). Overall, 52.4% of the clinicians reported using a clinical disease activity score. The Pouchitis Disease Activity Index (PDAI) was the most commonly used (196/412, 47.6%), whereas the Pouchitis Activity Score (PAS) was rarely employed (20/412, 4,8%). Nearly half (47.6%) reported not using any formal scoring system (Table 1).

**Table 1:** Clinicians’ perceptions and management practices regarding pouchitis in patients with IPAA (*N* = 412).

	Number of respondents over a total *N* = 412
**Estimated % of IPAA patients affected by pouchitis**	
**Less than 10%**	76 (18.4%)
**11%-25%**	120 (29.1%)
**26%-50%**	156 (37.9%)
**51%-75%**	56 (13.6%)
**More than 75%**	4 (1.0%)
**Average time after surgery when pouchitis occurs**	
**Less than 1 month**	6 (1.5%)
**1-3 months**	26 (6.3%)
**4-6 months**	110 (26.7%)
**7-12 months**	138 (33.5%)
**More than 12 months**	132 (32.0%)
**Follow-up frequency**	
**Once a month**	32 (7.8%)
**Every 3 months**	132 (32.0%)
**Every 6 months**	164 (39.8%)
**Every 12 months**	60 (14.6%)
**When they experience a disease flare-up**	24 (5.8%)
**Use of clinical scores to assess disease activity in IPAA patients**	
**No**	196 (47.6%)
**Yes, the Pouchitis Activity Score (PAS)**	20 (4.8%)
**Yes, the Pouchitis Disease Activity Index (PDAI)**	196 (47.6%)
**Yes, others**	0 (0%)

IPAA: Ileal Pouch-Anal Anastomosis.

#### Noninvasive monitoring in IPAA patients: Fecal calprotectin and intestinal ultrasound

Fecal calprotectin (FC) was the most widely used noninvasive monitoring tool (79.1%), with most clinicians assessing it every 3-6 months. However, the thresholds used to define remission varied considerably, with ≤150 µg/g being the most frequently selected cutoff (Figure 4).

**Figure 4:**
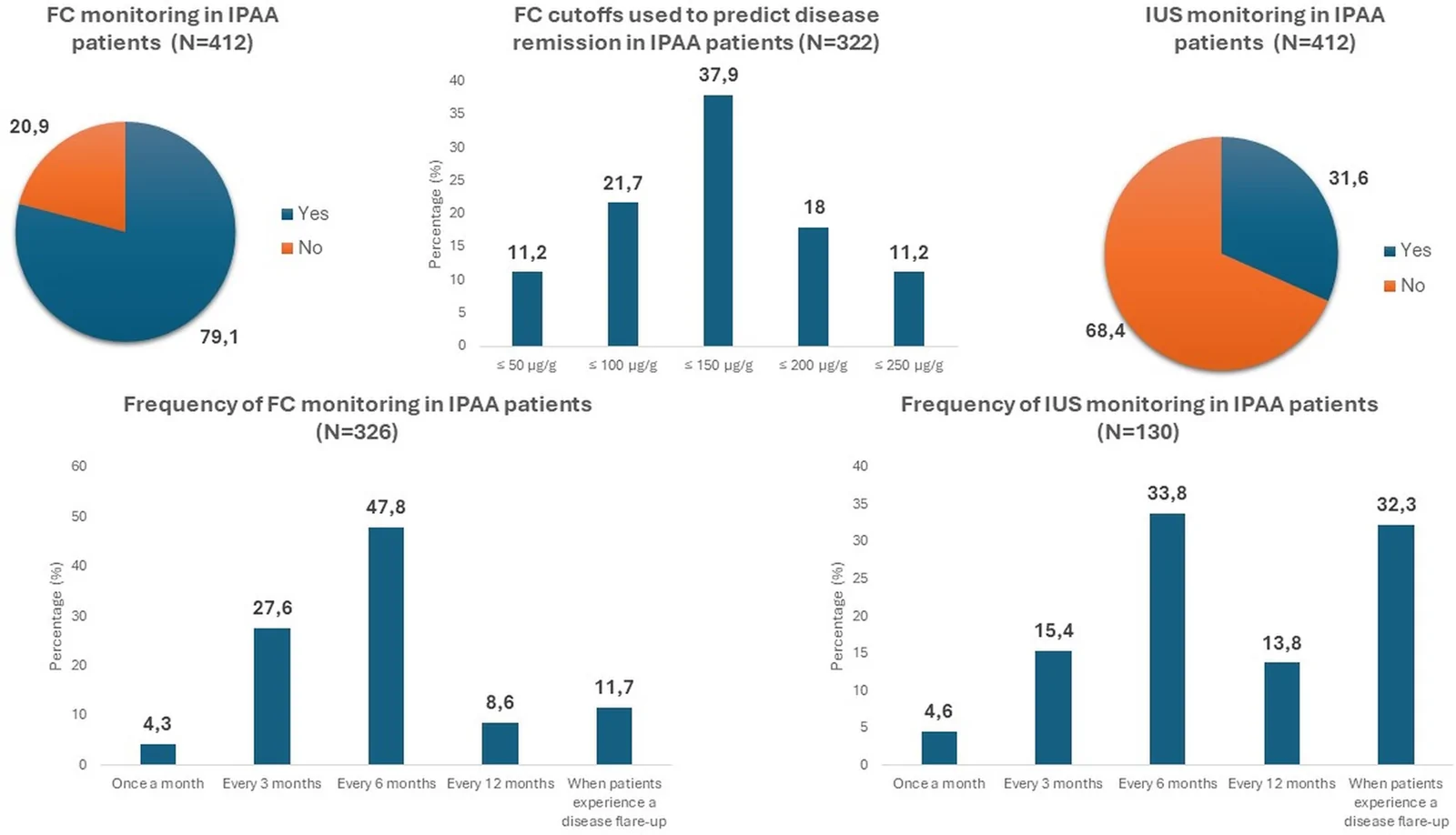
Noninvasive monitoring strategies in patients with IPAA.

Intestinal ultrasound (IUS) was less frequently used (31.6%). Among users, semiannual (33.8%) assessments and evaluations during disease flares (32.3%) were most frequent, while quarterly (15.4%), annual (13.8%), and monthly (4.6%) monitoring were less common (Figure 4).

#### Invasive monitoring in IPAA patients

Pouch endoscopy remained the cornerstone of IPAA monitoring, being reported by 96.6% of respondents. Annual surveillance and assessment during disease flares were the most commonly reported approaches, while the use of formal endoscopic scoring systems remained inconsistent (Figure 5).

**Figure 5:**
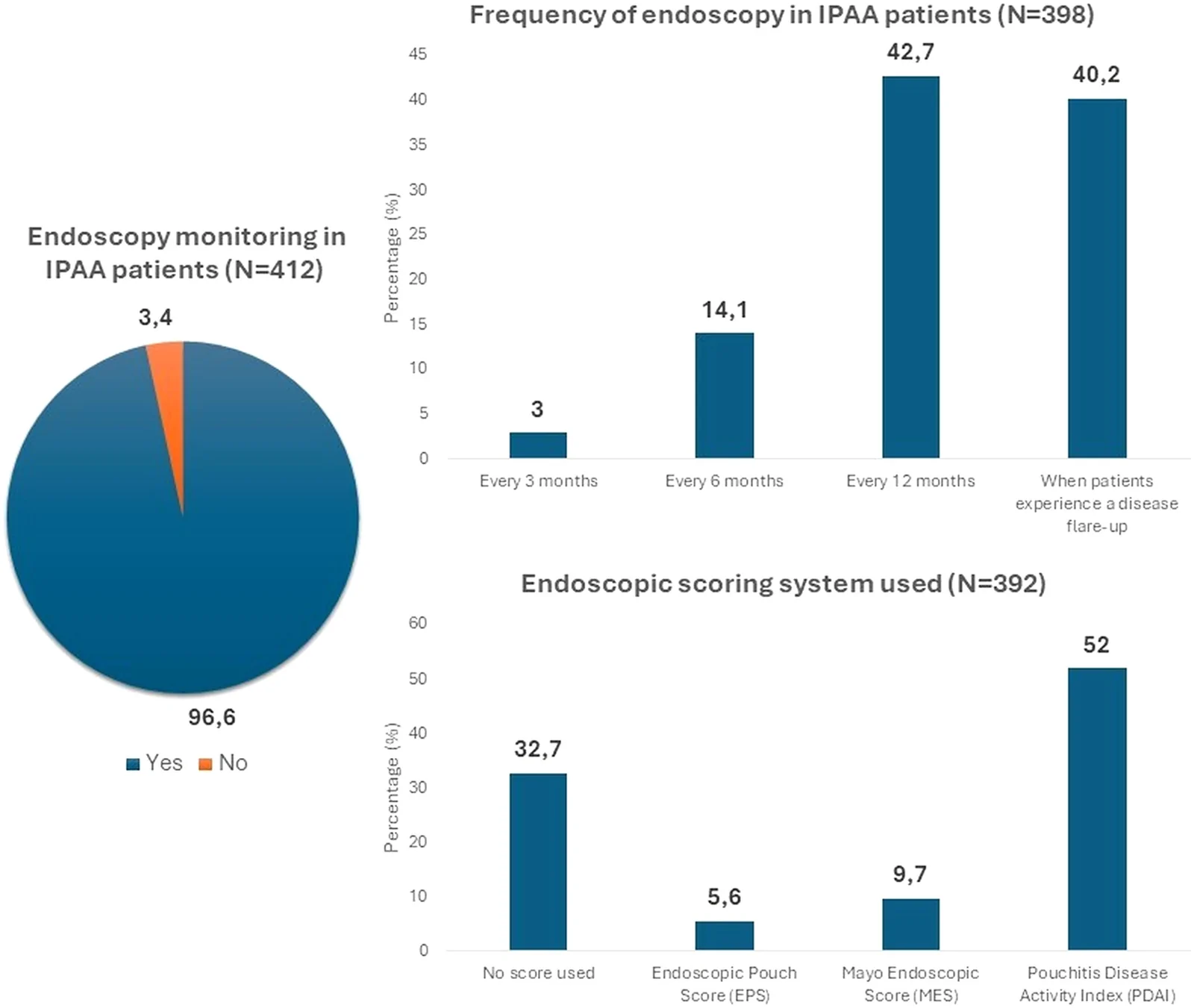
Invasive monitoring strategies in IPAA patients: Endoscopy.

Most clinicians (88.3%) reported performing biopsies to monitor histological activity in IPAA patients. Among those, when assessing histological activity, nearly half of respondents (46.7%) reported to not use any standardized scoring system. Among those who did, the Pouchitis Disease Activity Index (PDAI) (29.7%) and the Nancy Index (15.9%) were the most frequently adopted, while few reported using the PAS (7.1%).

Regarding the biopsy site, the pouch itself was the most common biopsy site reported (45.3%), followed by the pouch–anal anastomosis (24.3%) and other locations (25.4%), which frequently included cuff or afferent limb sampling. Biopsies from the neoterminal ileum alone were less frequently reported (5%) (Figure 6).

**Figure 6:**
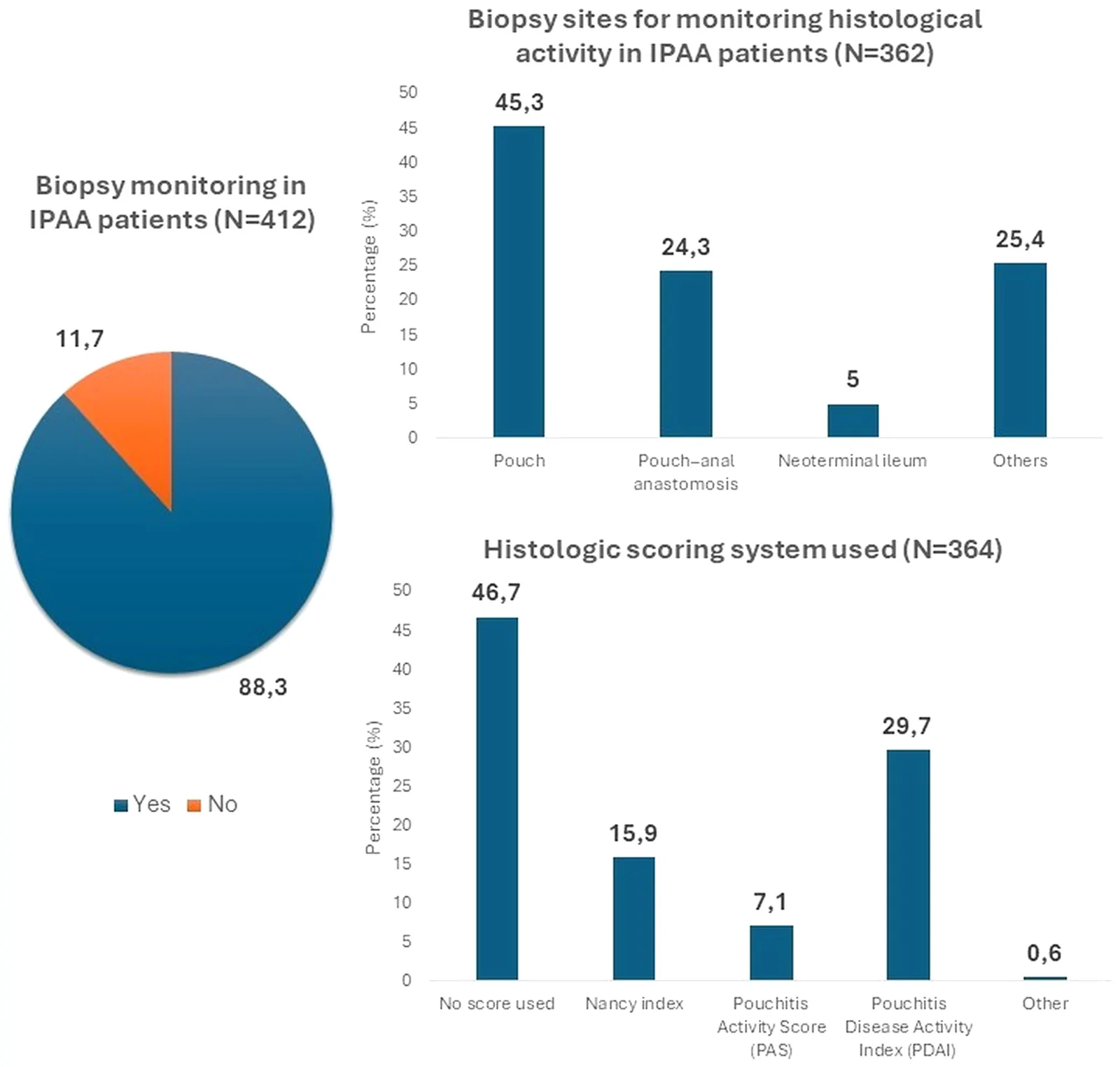
Invasive monitoring strategies in IPAA patients: Histology.

#### Variations in diagnostic and monitoring practices according to IPAA caseload and dedicated pouch clinic availability

The use of diagnostic and monitoring tools varied according to reported IPAA caseload and the availability of a dedicated pouch clinic. The use of clinical disease activity scores differed according to IPAA caseload, being reported by 55.7% of clinicians managing fewer than 10 IPAA patients annually, 44.8% of those managing 10-50 patients, and 62.5% of those managing more than 50 patients (*P* = .019). Similarly, the use of FC varied across IPAA caseload categories (82.3%, 69.0%, and 77.5%, respectively; *P* = .017), as did the use of formal endoscopic disease activity scores (64.6%, 58.6%, and 75.0%, respectively; *P* = .041).

The availability of a dedicated pouch clinic was also associated with differences in the use of objective disease assessment tools. Clinicians working in centers with a dedicated pouch clinic more frequently reported the use of clinical disease activity scores (64.7% vs. 48.4%; *P* = .006), IUS (35.3% vs. 16.1%; *P* < .001), formal endoscopic scoring systems (76.5% vs. 60.0%; *P* = .004), and standardized histological scores (58.8% vs. 42.6%; *P* = .006). Endoscopic remission was also more frequently identified as a treatment target among clinicians working in centers with a dedicated pouch clinic (39.2% vs. 27.1%; *P* = .029).

### Prevention and management of pouchitis

#### Prevention of pouchitis

Slightly more than half of the surveyed physicians (53.9%) reported prescribing medications to their IPAA patients to prevent the risk of pouchitis. The remaining clinicians did not report to routinely use prophylactic therapy. Among the 238 clinicians prescribing medications to prevent pouchitis in IPAA patients, a total of 570 responses were recorded, corresponding to an average of 2.39 therapeutic options selected per physician. Probiotics were the most frequently reported preventive strategy (27.7%), followed by rectal mesalazine (18.2%), and antibiotics such as metronidazole (17.9%) and ciprofloxacin (17.2%). Rifaximin and oral mesalazine accounted for 10.5% and 8.1% of the responses, respectively, while budesonide rectal foam rarely chosen (0.4%) (Figure 7).

**Figure 7:**
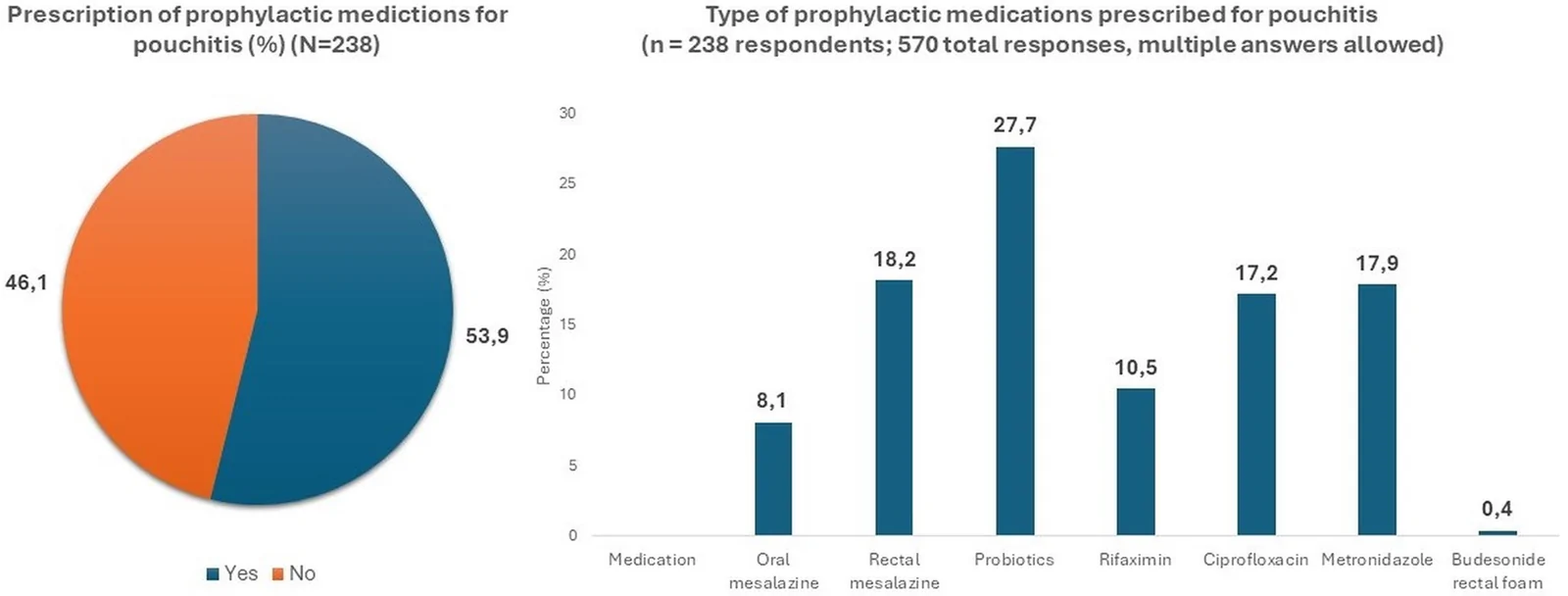
Medications prescribed to prevent pouchitis in IPAA patients.

#### Conventional treatment for pouchitis

The combination of ciprofloxacin and metronidazole was the most frequently chosen first-line treatment for pouchitis (52.9%). Ciprofloxacin alone was preferred by 27.2% of respondents, while metronidazole alone was selected by 19.4%. Only a very small minority used the amoxicillin–clavulanic acid combination (0.5%). The majority of surveyed clinicians reported prescribing antibiotics for 1-2 weeks when treating pouchitis (64.6%), followed by a substantial proportion who extend therapy to 4 weeks (24.8%). Shorter courses of less than 1 week (0.5%) or exactly 1 week (4.4%) or longer courses for more than 4 weeks (5.8%) were reported much less frequently.

When asked to define chronic antibiotic-dependent pouchitis, most clinicians identified the condition after 3-5 antibiotic cycles (68%), while approximately one third (29.1%) considered two cycles sufficient. Only a small minority defined the threshold as 5-10 cycles (2.9%), and no respondents defined it after a single cycle or more than ten cycles (Figure 8).

**Figure 8:**
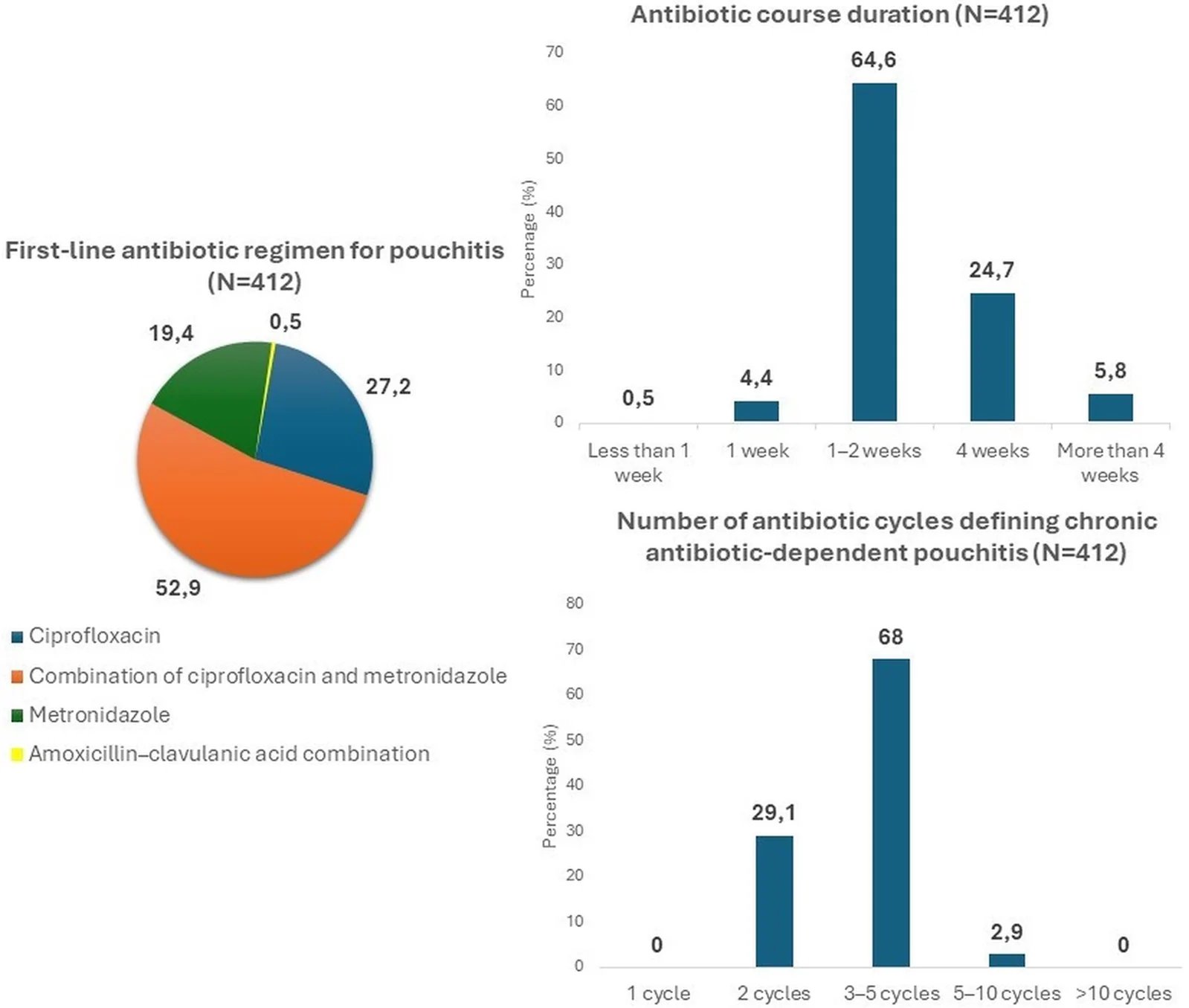
First-line antibiotic choices and treatment duration for pouchitis, definition of chronic antibiotic-dependent pouchitis.

### Advanced treatment for CARP and CADP

Advanced therapies were the most frequently chosen first-line treatment for patients with chronic antibiotic-dependent or refractory pouchitis (61.7%). Oral budesonide represented the second most common option (22.8%), followed by prednisone (10.2%) and other therapies such as beclomethasone (2.4%) or immunomodulators like thiopurines or methotrexate (2.9%). Among advanced therapies, vedolizumab (VDZ) (48.1%) and infliximab (IFX) (41.3%) were the most frequently selected first-line options in naïve patients, followed by adalimumab (ADA) (6.3%). Other agents including golimumab (GOLI), ustekinumab (USTE), mirikizumab (MIRI), etrasimod, ozanimod, tofacitinib, filgotinib, and upadacitinib were rarely selected as initial advanced therapies (Figure 9).

**Figure 9:**
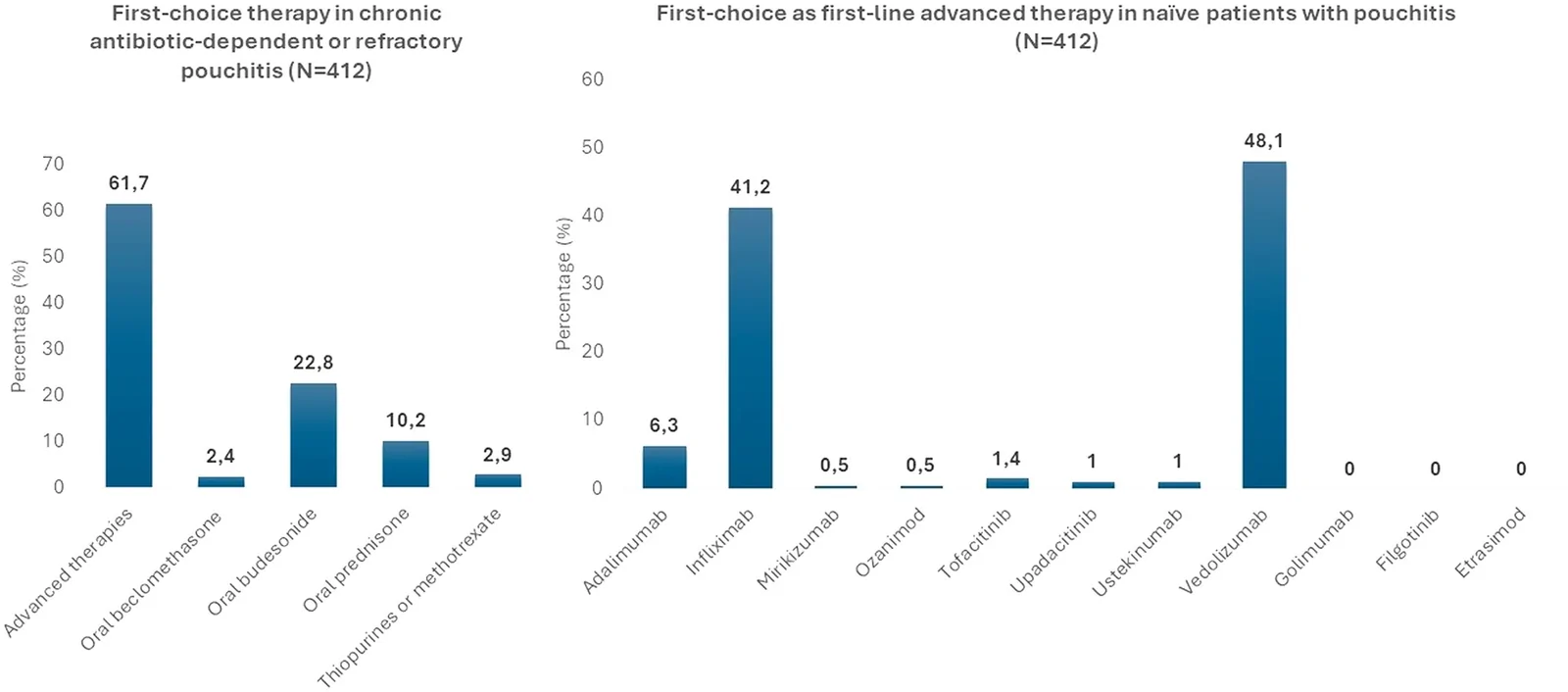
Therapeutic approaches and first-line advanced treatments for chronic antibiotic-dependent or refractory pouchitis.

The distribution of therapeutic choices across successive treatment lines revealed a clear hierarchy in the use of advanced therapies for pouchitis. IFX and ADA predominated in the early phases, being most frequently selected as first-line agents (172 and 150 responses, respectively), with a marked decline in use in subsequent lines. VDZ appeared mainly as second- to fifth-line option, reflecting its role in patients with prior anti-TNF exposure. In contrast, USTE and MIRI became increasingly prominent from the third to sixth lines, suggesting preferential use in refractory cases or after multiple biologic failures. S1P modulators and JAK inhibitors were predominantly selected in later treatment lines (Table 2).

**Table 2:** Sequencing of advanced therapeutic agents in the management of pouchitis (number of responses for each treatment line among 412 participants).

Therapy	1st line	2nd line	3rd line	4th line	5th line	6th line	7th line	8th line	9th line	10th line	11th line
**Infliximab**	172	30	6	198	0	0	0	0	2	0	4
**Adalimumab**	150	128	6	52	48	14	0	2	4	0	8
**Golimumab**	44	78	56	68	80	26	4	6	18	4	28
**Vedolizumab**	22	40	40	52	108	44	2	12	18	8	66
**Ustekinumab**	10	30	70	24	100	64	8	12	38	2	54
**Mirikizumab**	2	40	28	8	40	138	24	10	52	14	56
**Ozanimod**	8	18	52	2	20	46	110	26	46	38	46
**Etrasimod**	2	22	24	0	8	38	84	128	44	36	26
**Tofacitinib**	0	14	48	2	6	26	50	96	118	44	8
**Filgotinib**	0	6	26	2	0	12	70	78	60	146	12
**Upacitinib**	2	6	56	4	2	4	60	42	12	120	104

Most respondents (42.5%) reported that they would consider reusing a previously failed therapy if the patient had demonstrated a response before surgery. Approximately one-quarter (26.1%) would consider reuse only in the absence of alternative therapeutic options, while 19.5% considered absence of safety concerns as a prerequisite. In contrast, 11.9% of clinicians indicated that they would never reuse previously failed drugs (Table 3).

**Table 3:** Reuse of pre-surgery advanced therapies in pouchitis patients (total responses = 522, *n* = 412 participants, multiple answers allowed).

Response	Number of responses (%)
**No**	62 (11.9%)
**Yes, if the patient had a response**	222 (42.5%)
**Yes, if I do not have other options**	136 (26.1%)
**Yes, if there is no safety issue**	102 (19.5%)

When initiating advanced therapy for chronic pouchitis, clinicians may choose to continue or discontinue concomitant conventional treatments. In our survey, probiotics (23.18%) and rectal mesalazine (21.11%) were the most commonly selected therapies to be mantained, while systemic antibiotics (14.19%), oral mesalazine (7.61%), and rifaximin (5.54%) were less frequently selected. Among 412 participants, a total of 578 responses were recorded, corresponding to an average of 1.4 treatments continued per physician. Notably, over a quarter of respondents (26.64%) reported discontinuing all prior therapy when initiating advanced treatment. These findings suggest that clinicians often maintain selected topical or supportive therapies alongside advanced therapy, but a substantial proportion opts for a complete therapeutic reset (Table 4).

**Table 4:** Continuation of treatments when starting advanced therapy in patients with pouchitis (*n* = 412 respondents; 578 total responses, multiple answers allowed).

Medication		Number of responses (%)
**Oral mesalazine**		44 (7.61%)
**Rectal mesalazine**		122 (21.11%)
**Probiotics**		134 (23.18%)
**Rifaximin**		32 (5.54%)
**Antibiotics**		82 (14.19%)
**Don’t continue therapy**		154 (26.64%)
**Others**		
**Sulfasalazine**		2
**Rectal budesonide**		2
**Continuing conventional therapy previously used until the effect of advanced therapy is reached**		2
**Combination of antibiotics and probiotics**		4
		10 (1.73%)

### Treatment targets approach in patients with pouchitis

Clinical remission (37.9%) and endoscopic remission (30.1%) were the most frequently selected treatment targets in the management of pouchitis, whereas clinical improvement (18.4%) and endoscopic improvement (11.7%) were secondary goals. Normalization of FC was rarely selected (1.9%). Endoscopic monitoring was essential and routinely performed by the majority of clinicians (*n*  =  396/412, 96.1%). Nearly all clinicians scheduled endoscopic evaluation within the first 12 months of treatment initiation. Specifically, the majority (62.1%) reported scheduling the first endoscopic assessment at 3-6 months after treatment initiation, followed by 7-9 months (19.2%) and 12 months (7.6%). Early assessment within 3 months (9.6%) and delayed evaluation beyond 12 months (1.5%) were less frequently reported. In cases of loss of response to advanced therapy, most clinicians (59.7%) reported modifying the therapeutic regimen. However, a subset reported adjunctive approaches, including the use of antibiotics (14.6%), rectal corticosteroids (14.6%), or oral corticosteroids (9.2%) (Figure 10).

**Figure 10:**
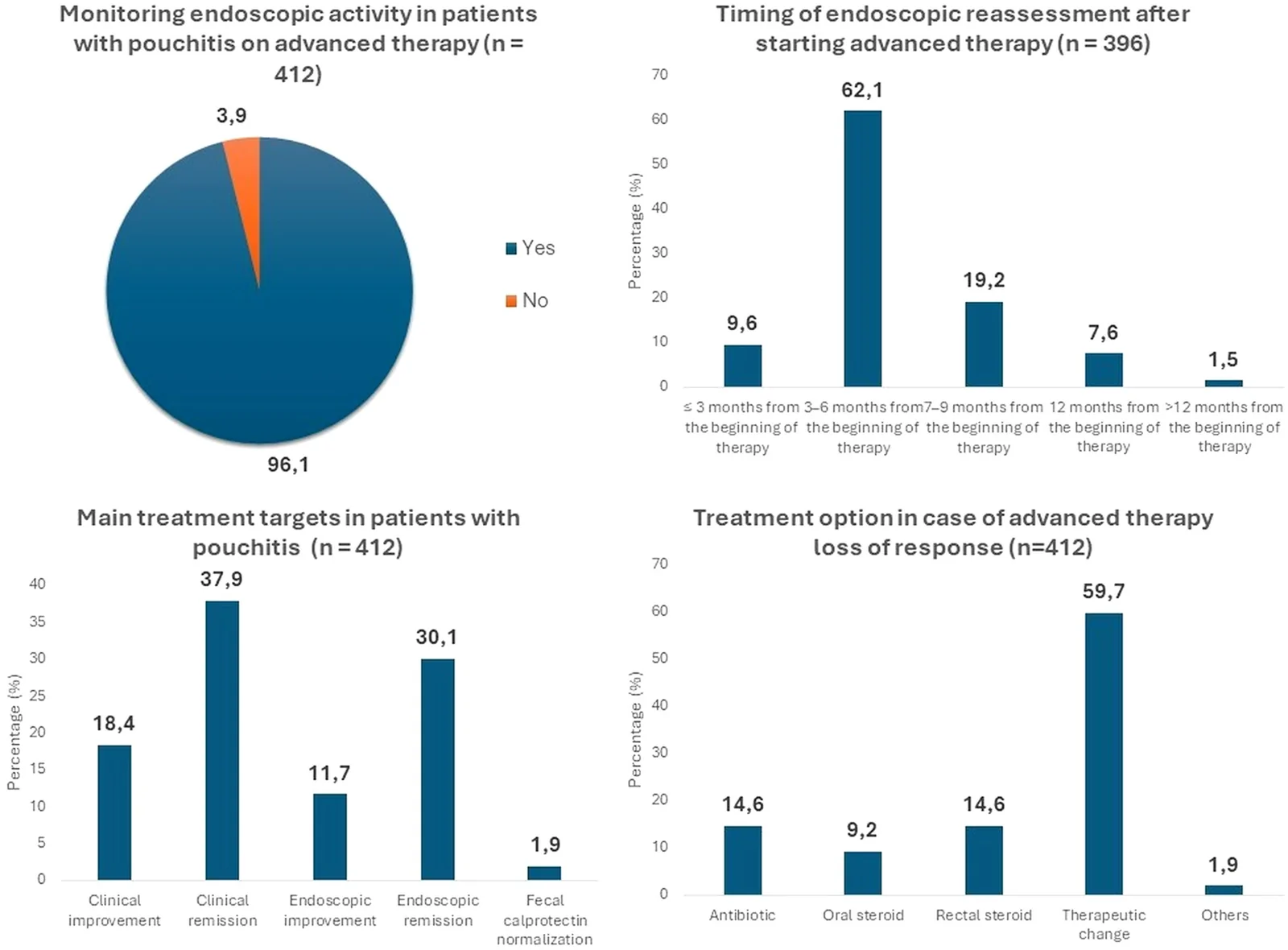
Treat-to-target management in patients with pouchitis.

### Pouch outcomes and complications

Nearly half of the surveyed clinicians (48.5%) estimated that pouch-related complications occurred in ≤5% of their patients, while approximately one-third (32.5%) reported rates between 6% and 10%. Only a minority (19%) observed complication rates above 10%, and none reported frequencies exceeding 50%. Overall, these findings indicate that pouch complications such as fistulas, stenosis, cuffitis, or malignancies are generally perceived as infrequent events in clinical practice.

More than half of the surveyed clinicians (52.9%) reported that ≤5% of their IPAA patients were ultimately reclassified as having Crohn’s disease (CD) rather than UC, while about one-third (30.1%) estimated rates between 6% and 10%. Similarly, pouch failure was reported in ≤5% of the patients by 52.4% clinicians and in 6%-10% by 31.1%. Only a small proportion (15%) described failure rates above 10%, with none reporting rates exceeding 50%.

In cases of pouch failure, permanent diversion with ileostomy was the most frequently adopted solution (55.3%). Redo-IPAA was selected by 17.5% of respondents, while 14.6% reported the use of dual advanced therapy and 5.8% long-term antibiotic maintenance. A small proportion (6.8%) reported other individualized approaches (Figure 11).

**Figure 11:**
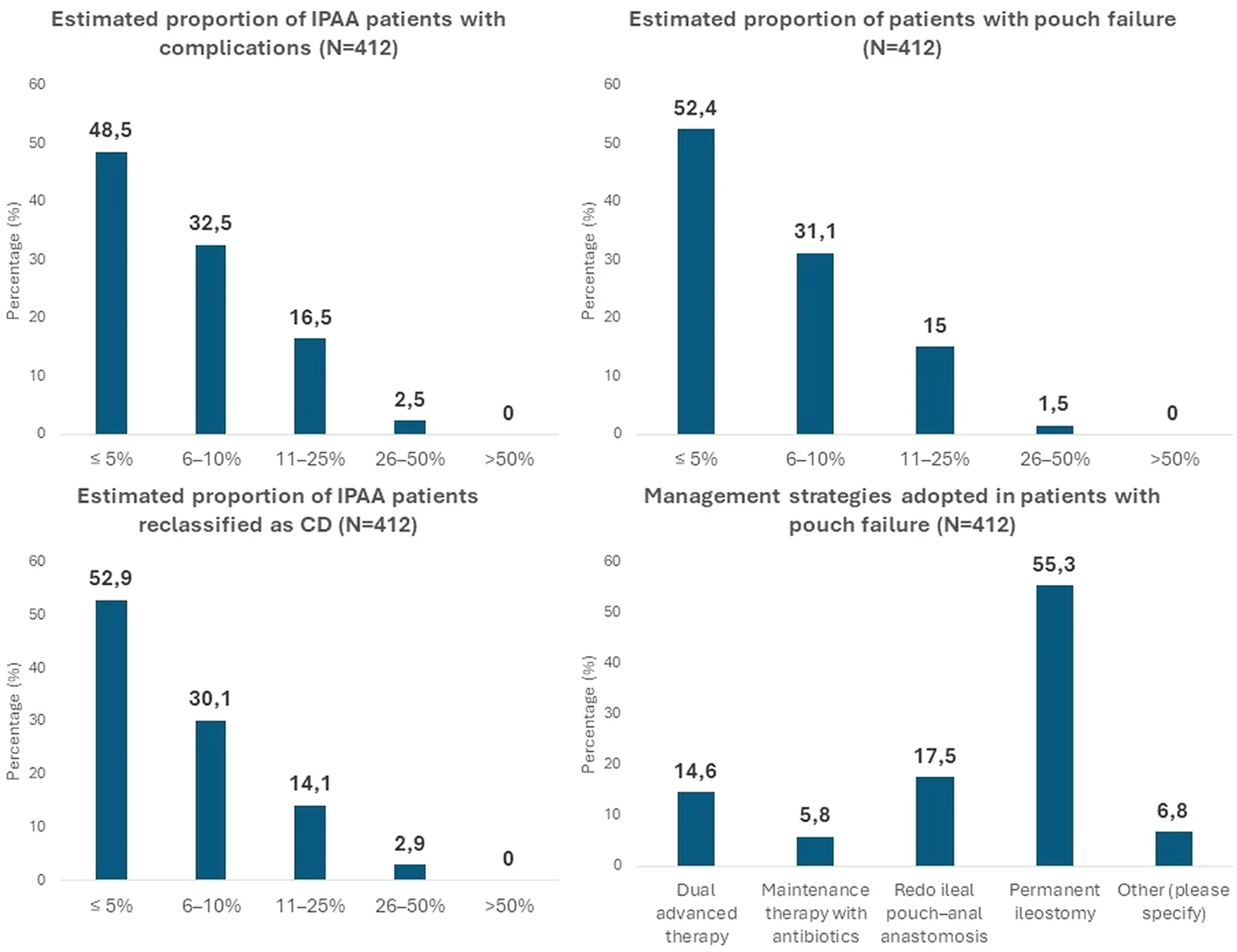
Estimated proportion of pouch outcomes and complications.

## Discussion

This international survey provides a comprehensive and detailed overview of contemporary real-world practices in the diagnosis and management of pouchitis among IBD specialists across diverse healthcare systems and practice settings. Importantly, the findings of this survey should be interpreted as a description of clinician-reported practice patterns rather than as measures of clinical effectiveness or patient outcomes. The observed differences between respondents and practice settings identify areas of heterogeneity and potential unmet needs, but do not establish that any specific diagnostic, monitoring, or therapeutic strategy is superior. Similarly, associations observed in the exploratory subgroup analyses should be considered hypothesis-generating and should not be interpreted as causal relationships. The marked heterogeneity observed across diagnostic definitions, monitoring strategies, and therapeutic approaches underscores the persistent lack of standardized, evidence-based care pathways for pouch disorders following IPAA and reflects the limited availability of high-quality, pouch-specific evidence. Variability in disease definition and assessment represents a major challenge. Although pouchitis is widely recognized as a common complication after IPAA, clinicians differ substantially in their estimates of disease prevalence, timing of onset, and criteria used to define chronic antibiotic-dependent disease. This heterogeneity may reflect differences in clinical experience, patient case-mix, and access to diagnostic tools, but also highlights the absence of universally accepted definitions and thresholds.^14^^,^^15^ The limited and inconsistent use of standardized clinical, endoscopic, and histological scoring systems further complicates objective disease characterization and response assessment. Although the PDAI remains the most frequently used instrument, existing pouchitis indices have not undergone comprehensive validation across all relevant measurement properties. The complexity of composite instruments, the time required for their implementation, and the absence of a universally accepted scoring system may contribute to their limited adoption in routine practice. This variability may hinder standardized disease assessment and treatment response evaluation, limiting comparability across studies and centers. These findings support the development and prospective validation of pragmatic, reproducible scoring systems that can be readily incorporated into routine clinical practice.^14^^,^^15^ Monitoring practices were similarly heterogeneous. FC was widely adopted as a noninvasive marker of pouch inflammation, with most clinicians favoring regular assessment every 3-6 months. However, the substantial variability in the thresholds selected by respondents highlights the lack of a standardized approach to FC interpretation in pouchitis. Although relatively low thresholds, particularly between 100 and 200 µg/g, were most frequently reported, no universally accepted cutoff has been established for the diagnosis, monitoring, or definition of remission in pouchitis. Previous studies have reported heterogeneous thresholds and variable diagnostic performance depending on the reference standard and disease definition.^16–18^ Consequently, the widespread use of FC in clinical practice is not yet accompanied by a standardized interpretation framework. Prospective studies incorporating paired FC, clinical, and endoscopic assessments are needed to establish clinically meaningful thresholds and determine whether FC-guided monitoring can reliably inform therapeutic decision-making.^16–18^ In contrast, IUS, despite growing interest in IBD, remains underutilized in IPAA patients, likely due to limited validation in this specific setting and the lack of pouch-specific ultrasound criteria.^19^ Invasive monitoring with pouch endoscopy remains central to disease assessment, particularly in patients receiving advanced therapies, with most clinicians performing endoscopic reassessment within the first year of treatment.^20^ Nevertheless, the wide variability in surveillance intervals and the inconsistent application of endoscopic and histological indices indicate that endoscopy-driven treat-to-target strategies have not yet been uniformly translated into routine pouchitis care. Interestingly, the availability of a dedicated pouch clinic was associated with greater use of clinical, endoscopic, and histological scoring systems, as well as IUS, and with more frequent selection of endoscopic remission as a treatment target. These findings suggest that specialized organizational structures may be associated with more systematic and objective disease assessment. However, because of the cross-sectional design of the survey, these associations cannot establish a causal effect of dedicated pouch clinics on clinical management or patient outcomes. Dedicated multidisciplinary pathways may nevertheless facilitate the integration of gastroenterological, surgical, endoscopic, radiological, and nursing expertise in the management of complex pouch disorders. Prospective studies are needed to determine whether such models can improve consistency of care and, ultimately, patient outcomes. Similarly, several diagnostic and monitoring practices varied according to reported IPAA caseload. Clinicians managing different numbers of patients with IPAA differed in their reported use of clinical disease activity scores, FC, and formal endoscopic scoring systems. These findings may suggest that greater exposure to pouch disorders is associated with differences in the adoption of structured assessment strategies. However, IPAA caseload may also reflect referral patterns, institutional expertise, and access to specialized resources, and the cross-sectional design does not allow these factors to be disentangled. These exploratory associations should therefore be considered hypothesis-generating rather than evidence of a direct effect of case volume on clinical practice. Therapeutic decision-making in chronic antibiotic-dependent and refractory pouchitis appears largely extrapolated from ulcerative colitis and Crohn’s disease rather than driven by robust, pouch-specific evidence.^20^^,^^21^ Advanced therapies were most frequently selected as first-line treatment in this setting, with vedolizumab and infliximab predominating. Importantly, vedolizumab is currently the only advanced therapy supported by randomized controlled trial evidence in chronic antibiotic-refractory pouchitis, as demonstrated by the EARNEST trial.^22^ This evidence base may partly explain the frequent selection of vedolizumab observed in our survey, although treatment choices may also reflect local experience, availability, and clinician preference. In contrast, the use of other biologics and small molecules appears largely guided by observational data, expert opinion, or prior experience in luminal IBD, contributing to the observed heterogeneity in therapeutic sequencing. The progressive shift toward alternative mechanisms of action in later treatment lines further highlights the challenges of managing refractory disease in the absence of validated treatment algorithms.^23^ Similarly, the selective reuse of previously failed therapies in some patients reflects a pragmatic and individualized approach, balancing prior efficacy, safety considerations, and therapeutic availability.^23^ Several limitations of this study should be acknowledged. First, the geographic distribution of respondents may affect the generalizability of the findings. Although the survey included clinicians from 49 countries, the sample was predominantly composed of European respondents, with Italy accounting for a substantial proportion of the overall sample. This geographic imbalance may have influenced the observed practice patterns and may limit the extrapolation of our findings to other healthcare systems and regions. Differences in healthcare organization, access to specialist expertise, availability of advanced therapies, reimbursement policies, and referral patterns may contribute to regional variation in pouchitis management. Therefore, the findings should be interpreted as an international snapshot of clinician-reported practice rather than as a fully representative estimate of global pouchitis management. No formal comparison of practice patterns across geographic regions was performed, given the uneven distribution of respondents across regions and the substantial predominance of European participants; therefore, regional differences should be interpreted cautiously. As a survey-based analysis, the findings are subject to recall bias and may reflect perceived rather than actual clinical practice. Voluntary participation may have introduced selection bias toward clinicians with a particular interest in pouch disorders or advanced therapies. In addition, an overall survey response rate could not be calculated because the survey was disseminated through multiple professional networks and mailing lists, and a reliable denominator of invited individuals was not available. This may further limit the assessment of the representativeness of the respondent sample. Importantly, the survey was not designed to assess patient-level outcomes and therefore cannot establish whether any of the reported diagnostic, monitoring, organizational, or therapeutic strategies are associated with improved clinical outcomes. Similarly, the exploratory subgroup analyses should be considered hypothesis-generating and cannot establish causal relationships. The relatively low frequency of pouch-related complications reported in our survey compared with previous studies may, in part, reflect differences in the recognition or reporting of individual complications, including cuffitis. Importantly, our survey did not assess cuffitis as an individual complication; respondents were instead asked to estimate the overall frequency of pouch-related complications, including fistulas, stenosis, cuffitis, and malignancy. Therefore, these data should not be interpreted as an estimate of cuffitis prevalence. Differences in disease definitions, diagnostic approaches, surgical techniques, follow-up duration, and the distinction between endoscopic findings and clinically relevant disease may further contribute to differences between clinician-reported practice patterns and published epidemiological estimates. Despite these limitations, the present study identifies important areas of practice variability and unmet need. The lack of standardized definitions, monitoring strategies, and treatment targets for pouchitis highlights the need for consensus-driven recommendations and greater incorporation of objective outcome measures into routine practice. Future research should prioritize prospective, pouch-specific studies to validate clinical, endoscopic, histological, and noninvasive assessment tools, establish clinically meaningful FC thresholds and treatment targets, and evaluate the comparative effectiveness of advanced therapies beyond vedolizumab. In parallel, the association between dedicated pouch clinics and more frequent use of structured disease assessment tools observed in our survey warrants prospective evaluation of multidisciplinary care models. Such studies may determine whether greater standardization and structured multidisciplinary care can reduce unwarranted variation in practice and ultimately translate into improved patient outcomes.^9^^,^^10^

## Conclusions

This international survey demonstrates substantial heterogeneity in the reported diagnosis, monitoring, and treatment of pouchitis, particularly in the use of standardized disease activity scores, noninvasive biomarkers, endoscopic assessment, and treatment targets. FC and pouch endoscopy are widely incorporated into clinical practice, but substantial variability remains in their interpretation and application. The availability of a dedicated pouch clinic and differences in IPAA caseload were associated with variation in the use of structured objective assessment tools, although these exploratory associations do not establish causality or differences in clinical outcomes. The geographic distribution of respondents, with substantial representation from Europe and Italy, should also be considered when extrapolating these findings to other healthcare systems. Overall, the survey highlights important areas of practice variability and supports the need for prospective studies and consensus-based approaches to standardize pouchitis assessment, monitoring and treatment.

## Data Availability

The data that support the findings of this study are available from the corresponding author, upon reasonable request.
